# The potential application of probiotics for the prevention and treatment of COVID-19

**DOI:** 10.1186/s43042-022-00252-6

**Published:** 2022-03-25

**Authors:** Engy Elekhnawy, Walaa A. Negm

**Affiliations:** 1 Pharmaceutical Microbiology Department, Faculty of Pharmacy, Tanta University, El-Geish Street, Medical Campus, Tanta, 31111 Egypt; 2 Pharmacognosy Department, Faculty of Pharmacy, Tanta University, Tanta, Egypt

**Keywords:** Antiviral, Beneficial microbes, Gut microbiota, Immunomodulatory, Probiotics

## Abstract

**Background:**

Given the severe infection, poor prognosis, and the low number of available effective drugs, potential prevention and treatment strategies for COVID-19 need to be urgently developed.

**Main body:**

Herein, we present and discuss the possible protective and therapeutic mechanisms of human microbiota and probiotics based on the previous and recent findings. Microbiota and probiotics consist of mixed cultures of living microorganisms that can positively affect human health through their antiviral, antibacterial, anti-inflammatory, and immunomodulatory effect. In the current study, we address the promising advantages of microbiota and probiotics in decreasing the risk of COVID-19.

**Conclusions:**

Thus, we recommend further studies be conducted for assessing and evaluating the capability of these microbes in the battle against COVID-19.

## Background

Respiratory infections could cause global high rates of morbidity and mortality. The viruses commonly associated with such infections include influenza viruses, parainfluenza viruses, coronaviruses, respiratory syncytial virus, rhinoviruses, and adenoviruses. In December 2019, an outbreak of pneumonia of unknown etiology was reported in Wuhan city in China [1]. WHO later identified this disease as Coronavirus disease (COVID-19) which is caused by a novel coronavirus called Severe Acute Respiratory Syndrome Coronavirus 2 (SARS-CoV-2). As yet, SARS-CoV-2 has become a global pandemic virus causing unprecedented crises regarding health, economy, and high mortality rate [2].

At present, there is no sole medication for the treatment of COVID-19 thus, researchers all over the world are actively engaged to find out appropriate treatment for COVID-19. Using beneficial microbe-based drugs could be a novel approach to be used in the attempts being done for treatment and prevention of COVID-19. The recent research about microbiota has led to an improved understanding of the communities of the commensal microorganisms (including bacteria, fungi, viruses, phages, archaea, and helminths) which live within the human body. Besides the extensively studied gut microbiota, the lung microbiota, which is only considered in recent years, represents an important member of the whole human microbiota [3]. It has been observed that in COVID-19 patients, there is microbial dysbiosis in the gastrointestinal tract (GIT) and lung which could be involved in the severity of the disease [4].

**Fig. 1:**
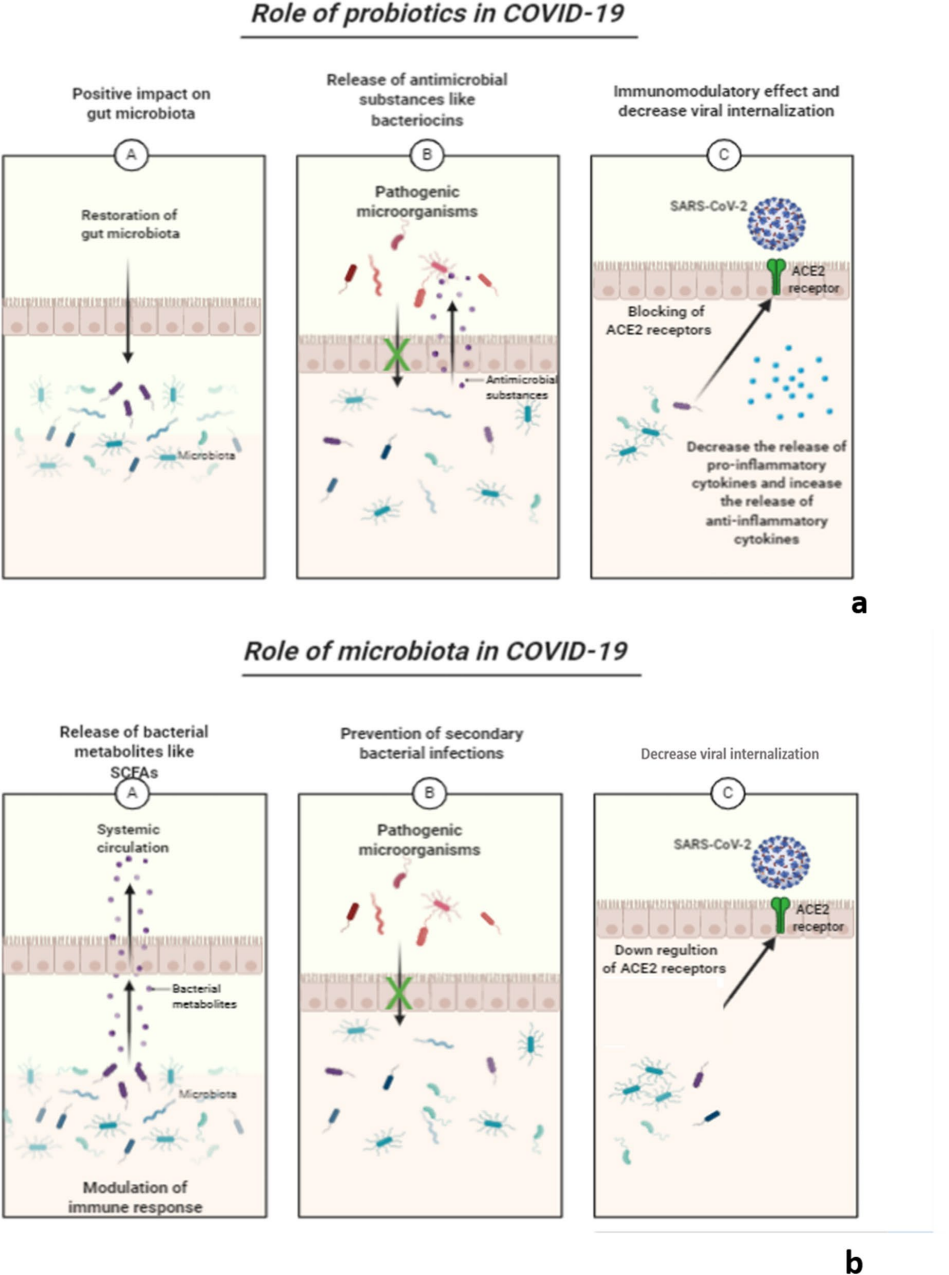
Role of **a** probiotics and **b** microbiota in treatment of COVID-19

Probiotics are defined as living microorganisms that, when given in appropriate amounts, afford beneficial effects to the host [5]. The potential of probiotics to boost health benefits has been reported as they can regulate allergic reactions, alleviate inflammatory bowel disease, reduce tumor growth in some cancer models, prevent colon cancer, control the levels of blood cholesterol and protect hosts from bacterial and viral infections [6]. Human microbiota and probiotics have anti-inflammatory and immunomodulatory effects that could be beneficial in the treatment of the severely ill COVID-19 patient who always suffers from cytokine storm that results from the production of a large quantity of pro-inflammatory cytokines [7]. Besides, they have an antiviral and antibacterial activity which is necessary for our fight against the SARS-CoV-2 virus [8, 9]. The rationale of using biotherapeutic drugs based on beneficial microbes like human microbiota and probiotics for treatment and prevention of COVID-19 infection is attributed to their antiviral, anti-inflammatory, and immunomodulatory effect, they also can prevent secondary bacterial infections as presented in Fig. 1.

## Main text

### Gut-lung axis and COVID-19

The alimentary tract hosts a complex group of the highly diverse microbial ecosystem which has a role in ensuring the establishment and persistence of immune homeostasis [10]. In addition to the widely investigated gut microbiota, the microbiota of other sites in the human body, especially the lungs, are crucial for host homeostasis. Interestingly, lung microbiota is now recognized to have an essential role in the physiopathology of many respiratory diseases [11]. Consequently, a group of researchers has investigated if the infection caused by SARS-CoV-2 affects the lung microbiota [12]. They observed a severe microbiota dysbiosis in the lungs of COVID-19 patients, with a high incidence of pathogenic species like *Klebsiella oxytoca* and Tobacco mosaic virus (TMV) a finding which could contribute to the complications that occur in SARS-CoV-2 infections. From birth and throughout the entire life span, a close correlation exists between the gut and lung microbiota [13]. For example, if the newborns’ diet is modified, the composition of their lung microbiota will be affected, and fecal transplantation in experimental rats can induce changes in their lung microbiota [3].

On the opposite side, the lung microbiota could affect the gut microbiota. In an experimental model, Looft and Allen [14] found that infection with influenza virus triggers an increased abundance of *Enterobacteriaceae* and decreased proportions of *Lactobacilli* in the gut. This connection is called the gut-lung axis and the mechanisms mediating this communication are still unclear [15]. Although respiratory distress is a main symptom of COVID-19, this disease is also associated with some other non-classical symptoms such as gastrointestinal symptoms. Noteworthy, patients with gastrointestinal symptoms had more serious respiratory complications. This could be associated with microbial dysbiosis in the lungs and GIT [16].

### Human microbiota-virus interaction

Substantial interactions occur between the viruses invading the human body and commensal microbiota leading to certain suppressive outcomes for the viral infection [17]. This is based on the research carried out by Botic et al. who noticed that lactic acid bacteria (LAB) [18], which colonize the human gut, decreased the infectivity of vesicular stomatitis virus by direct binding to the virus, thus they blocked the entry of the viruses to the human cells. Also, Wang and his colleagues showed that *Enterococcus faecium*, a Gram-positive bacterium living in the human GIT, can prevent the influenza viral infection by direct adsorptive trapping of the viruses [19]. Furthermore, human microbiota can exert antiviral activity by its cellular components or through the production of several metabolites with antimicrobial activity [17].

An extracted cell wall-associated component from *Lactobacillus brevis* vaginal strain is an example of the antiviral activity of the microbiome cellular components. It has been found that this component potently inhibited the HSV-2 viral replication in an in vitro model [20, 21]. On the other hand, the extracellular matrix-binding protein which is produced by *Staphylococcus epidermidis* (bacterial commensal found in the human nasal cavity) can stably bind to the influenza virus thus, blocking further viral infection [22]. Microbiota may also have a role in decreasing the entry of SARS-CoV-2. It is well known that the SARS-CoV-2 virus enters human cells by transmembrane spike glycoprotein forming homotrimers expressed on its surface. This spike glycoprotein binds to trans-membrane angiotensin-converting enzyme (ACE2) receptor, which is expressed in different tissues in the human body like lung, kidney, and GIT [23]. Yang and his colleagues [24] studied the effect of microbiota on colonic ACE-2 receptors in a murine model and they noticed that gut microbiota regulated these receptors. Notably, various research articles have speculated the interaction of microbiota with ACE2 receptors in certain diseases like cardiovascular diseases [25] and intestinal inflammation [26, 27].

### Anti-inflammatory and immunomodulatory effect of human microbiota

An indirect role of microbiota on viral infection is its anti-inflammatory and immunomodulatory effect. Microbiota, especially gut and lung microbiota have effects on the local immunity [28, 29]. Gut microbiota can trigger the local immune response through interactions with the immune cells expressing pattern recognition receptors (PRRs) (e.g., Toll-like receptors [TLRs]) [30]. They can also activate local dendritic cells through interactions with PRRs [31]. Then the activated dendritic cells travel from the GIT to mesenteric lymph nodes, where they induce the differentiation of the T cells into the effector T cells, mainly regulatory T cells (Tregs) and T helper 17 (Th17) cells. Some of these effector T cells migrate back to the GIT and affect the local immune responses [32]. Tregs can mediate the conversion of the immune system from the pro-inflammatory to the anti-inflammatory state via the release of anti-inflammatory cytokines (like IL-10, TGF-β) [33].

Besides, several microbiota-derived metabolites like short-chain fatty acids (SCFAs) were found to protect the integrity of the GIT barrier against the disrupting effects of the pro-inflammatory cytokines [34]. A vital role of the lung microbiota in both maturation and homeostasis of lung immunity has been revealed over the last few years [29]. Preclinical studies confirmed the impact of lung microbiota on the regulation and maturation of immune cells of the respiratory system [34–37]. On the other side, the gut microbiota has a long-reaching immune impact (systemic effect), mainly on the pulmonary immune system via the mesenteric lymphatic system through which the intact microbiota, their fragments, or metabolites (like SCFAs) may reach the systemic circulation and modulate the immune response of the lung [38]. Many researchers have studied the immunomodulatory impact of SCFAs impact on the pulmonary system [39–41]. They noticed that SCFAs act as signaling molecules on the antigen-presenting cells of the lungs leading to attenuation of the inflammatory and allergic responses. Yin and his colleagues [42] have conducted bacteria research on the segmented filamentous (SFB), members of the gut microbiota, in humans and mice and they noticed that SFB has a significant role in the modulation of the host immune systems.

### Microbiota and prevention of secondary bacterial infections

One more important role of the human microbiota is colonization resistance where commensal microbiota protects the host against colonization with pathogenic organisms and inhibits the overgrowth of the pathogenic microbiota members. The postulated mechanisms of action for colonization resistance are: (1) directly by the interaction between human microbiota and different pathogens in competition for the shared niches and nutrients, and (2) enhancement of the host defense ability by the human microbiota to suppress pathogens (as discussed before). The dominant non-pathogenic microbiota plays an important role in both occupying the niche and inhibiting the colonization and growth of different pathogens [33]. Yet, if microbiota is disturbed for any reason, a decrease in the non-pathogenic dominant microbiota members decreases the capacity of colonization resistance, leading to colonization and overgrowth of the opportunistic pathogens in the empty niches. A classic example of this situation is the infection with *Clostridium difficile* which can cause pseudo-membranous colitis, sepsis, and death in severe cases [43].

### Fecal microbiota transfer as an example for microbiome-based biotherapeutic drug

Fecal microbiota transfer (FMT) involves the suspension of the donor stool in certain solutions, homogenization then filtration and finally, it is delivered through upper and/or lower GIT as gelatin capsules after centrifugation [44]. FMT is approved as a therapy for the treatment of recurrent infection with *Clostridium difficile* [45, 46]. It is now under research to be used in the treatment of some other diseases like metabolic disorders [47] and hepatic encephalopathy [48]. The main benefit of the use of FMT is to restore gut health and to reverse the gut dysbiosis that is induced by either antibiotic [49] or microbial infection [50] like in the case of COVID-19 infection. Thus, depending on the previously mentioned associations between gut microbiota and respiratory diseases, FMT could be effective in the treatment of COVID-19 patients.

### Immunomodulatory effect of probiotics

The effectiveness of probiotics in the treatment and prevention of a variety of diseases have been investigated like the prevention of allergy and certain intestinal diseases, in addition to the treatment of gastrointestinal diseases and certain types of cancers [51]. The health benefits conferred from probiotics are attributed to their effects on the immune system. Immunomodulators can be classified into immunostimulants or immunosuppressants [52]. The immunomodulatory effect of probiotics has been identified via the release of cytokines, interleukins, interferons, transforming growth factors, tumor necrosis factors (TNF), and chemokines from different immune cells such as lymphocytes, macrophages, mast cells, epithelial cells, granulocytes, and dendritic cells which boost the regulation of innate and adaptive immune system [53]. Several types of genera of bacteria have been identified as probiotics, among them, *Lactobacillus* and *Bifidobacterium* have been consumed as a part of fermented foods like those in dietary supplements [54]. It was found that *L. reuteri* and *L. casei*, can stimulate the production of IFN-gamma, and activate the pro-inflammatory Th1 cells [55]. Also, the oral administration of *B. infantis* into mice was noticed to stimulate dendritic cells that can suppress the biased responses of Th2 cells and stimulate the pro-inflammatory responses of Th1 that are required for virus elimination [56]. Besides, probiotics *L. acidophilus, L. gasseri*, *L. delbrueckii,* and *B. bifidum* strains can induce the production of IFN-alpha by monocytes [57]. The probiotic *L. paracasei* was found to increase the release of TNF-alpha, IL-6, IL-8 of the human monocyte cell line that is required for the antiviral effect [58].

### Antiviral activity of probiotics

A growing interest in the effectiveness of probiotics as viral inhibitors has emerged in the treatment of diseases and infections associated with HIV [59]. Probiotics have exhibited a potential role as antiviral agents against several groups of viruses like rotavirus [60], coxsackieviruses, enterovirus, [61], and herpes simplex [62]. Interestingly, the exo-polysaccharides produced by *Lactobacillus plantarum* were shown to have an antiviral effect against human rotavirus-induced diarrhea [63] and transmissible gastroenteritis virus [64]. Probiotics have antiviral activity against many respiratory viruses like influenza and syncytial viruses via boosting the immunity of individuals through activating the secretion of IgA and enhancing the activity of neutrophils, natural killer cells, and macrophages [65, 66].

### ACE inhibitory activity of probiotics

As previously mentioned, the entry of SARS-CoV-2 is facilitated by binding to ACE2 receptors and this interaction, when occurs in the gut, may be responsible for the GIT symptoms, which are reported in 12–60% cases of COVID-19 and it could be associated with increased disease severity [67]. In an interesting study [68], four metabolic products of *Lactobacillus plantarum*; Plantaricin BN, Plantaricin W, Plantaricin D, Plantaricin JLA-9 have been selected to design computer-based antiviral computational product for COVID-19. This study aimed to target and block the residual binding protein (RBP) on ACE2 receptor proteins by selected probiotics along with RNA-dependent RNA polymerase (RdRp). Three metabolic products of *L. plantarum*, significantly interacted with RdRp and ACE2, recording the lowest binding energy. These results suggest that probiotics could be used as a potential ACE2 receptor blocker, hence their importance in treating COVID-19 [68].

### Antimicrobial substances produced by probiotics

Probiotics like LAB can produce antimicrobial substances such as bacteriocins that have a broad spectrum of antagonistic effects against many bacterial pathogens [69]. Bacteriocins have been considered as promising antimicrobial compounds with potential applications in the food, health, and veterinary sectors [70]. Novel applications of LAB bacteriocins are steadily increasing, with horizons of more fascinating roles to be played by these agents in the future in anti-quorum sensing strategies and site-specific drug delivery [71]. Additionally, LAB strains often produce polymeric substances such as exopolysaccharides (EPS) that are proven by several researchers to have the ability to express antagonistic effects against pathogenic bacteria [72–77]. LAB can also produce biosurfactant agents which have shown a broad range of antimicrobial activity against bacterial pathogens as well as anti-adhesion properties that can reduce the pathogens' adhesion to the gastric wall membrane [69]. This ability of probiotics is important to fight against the secondary bacterial infections that commonly occur in severely ill COVID-19 patients.

### Impact of probiotics on gut microbiota and its link with COVID-19

Probiotics exert their beneficial effects via various mechanisms including treatment and restoration of gut microbiota, enhancement of intestinal barrier function, competition with pathogens for adhesion to gut epithelium and nutrition, suppression of opportunistic pathogens, production of antimicrobial substances, activation of mucosal immunity, and modulation of the innate and adaptive immune response. These actions of probiotics have been proven in various experimental and clinical studies [78]. As early mentioned, the respiratory viral infection is known to cause a disturbance in the gut microbiota, as in cases of COVID-19 infection, the gut microbiota is altered with severe hypoxemia. Some probiotic strains may restore the gut microbiota, maintain a healthy gut-lung axis, reduce translocation of pathogenic bacteria across gut mucosa and reduce the chances of secondary bacterial infection [79]. The most commonly used species in probiotics preparations are *Lactobacillus* sp, *Bifidibacterium* sp, *Enterococcus* sp, *Streptococcus* sp, *Bacillus* sp, and *Pediococcus* sp. Table 1 illustrated examples of different probiotics microbes, mechanisms of action, and their beneficial health effects [80–83]. It was reported that most of the patients with relatively mild symptoms of COVID-19 had received probiotics along with the established treatment protocols and this is in agreement with COVID-19 infection affecting the normal bacterial balance in the human intestine based on the observation of reduced numbers of *Lactobacillus* and *Bifidobacterium* species in patients with COVID-19 [84].

**Table 1:** The commonly used species in probiotics, mechanisms of action, and effects

Probiotics	Examples	Mechanism	Beneficial health effects
*Lactobacillus* sp	*L acidophilus* *L delbrueckii subsp bulgaricus* *L casei* *L cellobiosis* *L fermentum* *L curvatus* *L returi* *L plantarum* *L brevis* *L lactis*	Increasing mucin production via Increased expression of MUC 2	Improved mucosal immune function, mucin secretion, and disease prevention Adhering to human intestinal cells and balancing intestinal microflora mproved lactose digestion and decreased diarrhea
*Bifidibacterium* sp.	*B bifidum* *B adolescentis* *B thermophilum* *B animalis* *B infantis* *B longum*	Cytokine production Blocking proinflammatory	Used in treating rotavirus diarrhea, balancing intestinal microflora, and treating viral diarrhea
*Enterococcus* sp.	*E faecalis* *E faecium*	Prevention of pathogenic strains from adhering to epithelial cells	Decreased duration of acute diarrhea from gastroenteritis
*Streptococcus* sp.	*S cremoris* *S salivarius* *S diaacetylactis* *S intermedius*	Immune modulation by attenuating IL-8 secretion or blocking the degradation of the counter-regulatory factor IκB	Shortening of duration of acute gastroenteritis Prevention and treatment of *C difficile* diarrhea and traveler’s diarrhea
*Bacillus* sp.	*B licheniformis* *B subtilis* *B polyfermenticus* *B coagulans* *B laterosporus* *B polymyxa* *B pumilus* *B clausii* *B cereus var toyoi*	Enhanced antibody production Enhanced phagocytic activity	Used as a prophylactic, and in prevention of GIT infections
*Pediococcus* sp.	*P acidilactici*	Blocking proinflammatory molecules Increasing mucosal immunity	Enhanced immune responses against infectious coccidioidal diseases

## Conclusion

Based on the aforementioned impacts of both microbiota and probiotics, we strongly believe that microbiota and probiotics-based drugs have antiviral potential which deserves more investigation of their role in the prevention and treatment of COVID-19. Preclinical and clinical trials should be carried out in the near future to get benefits from these beneficial bacteria in the treatment of COVID-19 pandemic. In addition, the COVID-19 prevention guidelines should include these bacteria as an important means to fight against COVID-19 infection. We will be so excited to see how they will be applied in the clinical practice and afford therapeutic benefits to patients and high-risk individuals.

## Data Availability

Not applicable.

## Abbreviations

SARS-CoV-2: Severe Acute Respiratory Syndrome Coronavirus 2
TMV: Tobacco mosaic virus
LAB: Lactic acid bacteria
SCFAs: Short-chain fatty acids
ACE2: Angiotensin-converting enzyme
TNF: Tumor necrosis factors
PRRs: Pattern recognition receptors
FMT: Fecal microbiota transfer
RdRp: RNA-dependent RNA polymerase
GIT: Gasterointestinal tract
MUC 2: Mucin
